# Mechanism of action of decitabine in treating acute lymphoblastic leukemia

**DOI:** 10.3389/fmed.2025.1614592

**Published:** 2025-07-30

**Authors:** Xiaohui Gao, Hui Zeng, Fei Sun, Xiaoyan Zhao, Haibing Wu, Minchao Yan, Yuan Li, Qinyan Fu, Gang Zhang

**Affiliations:** ^1^Department of Pediatrics, The Affiliated Hospital of Jiaxing University, Jiaxing, Zhejiang, China; ^2^Department of Hematology, The Affiliated Hospital of Jiaxing University, Jiaxing, Zhejiang, China

**Keywords:** decitabine, acute lymphoblastic leukemia, PTEN, 4EBP1, tumor growth

## Abstract

**Purpose:**

This study aimed to evaluate the underlying mechanisms of decitabine (DAC) in inhibiting acute T-acute lymphoblastic leukemia (T-ALL) cell proliferation and promoting apoptosis.

**Methods:**

Human T-ALL cells (CCRF-CEM) were treated with varying concentrations of DAC, and cell proliferation was assessed using a CCK-8 assay. Flow cytometry was used to detect apoptosis and cell cycle alterations. The expression levels of apoptosis-related genes, including *PI3K* and *miR-92b-3p*, were quantified using real-time PCR (RT-PCR). Western blotting was used to analyze the expression of apoptotic proteins. Furthermore, we evaluated the *in vivo* antileukemic activity of DAC using a nude mouse xenograft model, monitored the body weight and tumor volume of mice to calculate inhibition rates, and examined tumor morphological changes in histological sections.

**Results:**

DAC significantly inhibited the proliferation of CCRF-CEM cells, accelerated apoptosis, and effectively downregulated the expression of PI3K, AKT, 4EBP1, and mTOR while concurrently upregulating PTEN protein expression. Its regulatory efficacy was markedly enhanced by increasing the dosage. Animal experimental results indicated that both DAC and doxorubicin substantially decreased tumor length, width, volume, and mass; however, DAC demonstrated significantly superior efficacy in inhibiting tumor growth compared to doxorubicin.

**Conclusion:**

By selectively targeting the regulation of PTEN and 4EBP1, along with their associated downstream signaling pathways, DAC effectively modulated cellular proliferation, facilitated apoptotic processes, and restrained tumor growth, providing a robust theoretical foundation for clinical treatment strategies in T-ALL.

## Introduction

Acute lymphoblastic leukemia (ALL) is a malignant tumor of the blood and immune system characterized by the abnormal proliferation of immature lymphocytes. It is most prevalent in children, comprising approximately 80% of acute leukemia cases. Cure rates vary significantly by country, reaching approximately 90% in high-income nations but dropping to 40–60% in low/middle-income countries due to limited access to intensive chemotherapy and supportive care (1–3). Childhood ALL is categorized into B-cell (85%) or T-cell precursor types. T-ALL accounts for nearly 15% of cases, exhibits a worse prognosis than B-ALL due to distinct molecular features and limited therapeutic options, highlighting the need for targeted strategies. This disease predominantly occurs in children aged between 1 and 4 years old, after which the incidence rate declines significantly (4). Although the exact causes of ALL remain unclear, several risk factors have been identified, including environmental factors such as exposure to ionizing radiation, certain chemical substances, and infection with HTLV-1. Individuals with somatic chromosomal translocations such as t(9;22), and those with germline genetic disorders including Down syndrome, Bloom syndrome, or neurofibromatosis, are at elevated risk. Down syndrome, caused by trisomy 21, is a germline chromosomal abnormality, while t(9;22) (BCR-ABL1 fusion) arises somatically in hematopoietic cells (5, 6).

Currently, while treatment for ALL has seen some advancements, managing therapy according to strict medication schedules poses challenges; fluctuations and recovery patterns of blood cell counts are difficult to predict precisely, leading to unforeseen, severe infections and other adverse effects known as periods of cytopenia (7). To address this challenge, frequent adjustments to chemotherapy doses and regimens based on empirical evidence and individual patient responses have become common practice, particularly for drugs that can cause bone marrow suppression, with the aim of minimizing cytopenia and infection risk (8). However, nonstandardized treatment modifications may undermine therapeutic efficacy, highlighting the urgent need to explore new therapeutic targets to optimize treatment outcomes and enhance clinical effectiveness in patients with ALL (9).

Decitabine (DAC) is an FDA-approved, 2-deoxycytidine analog used to treat myelodysplastic syndrome. As a demethylating agent, DAC facilitates leukemia cell differentiation and cancer cell apoptosis while inhibiting cell proliferation at low doses. Integrating DNA and depleting DNA methyltransferases induces cytotoxicity and suppresses DNA synthesis at higher doses, thereby exhibiting a dual mechanism of action (10, 11). DAC demonstrates a potent antileukemic potential by reversing DNA hypermethylation and activating dormant tumor suppressor genes. It has emerged as a pivotal component in bridging pretransplant therapy with posttransplant maintenance strategies, effectively preventing disease relapse (12–14). Studies have indicated that DAC, by enhancing the expression of HLA-DR and FoxP3, can concurrently inhibit GVHD and preserve the graft-versus-leukemia effect, thereby demonstrating its potential as a novel and efficacious treatment for relapse following allo-HSCT (15, 16). This study aimed to evaluate the apoptotic effect of DAC on CCRF-CEM leukemia cells and further investigate its impact on cell proliferation and apoptosis induction. Key genes were selected based on their established roles in the PI3K/AKT/mTOR pathway, a central regulator of cell survival and apoptosis (17). *PI3K* and *AKT* are positive regulators of cell proliferation, while *PTEN* acts as a tumor suppressor by inhibiting PI3K (18, 19). *4EBP1*, a downstream target of mTOR, regulates protein synthesis and apoptosis when dephosphorylated (20). Additionally, a xenograft mouse model was used to assess the *in vivo* anti-ALL activity of DAC.

## Materials and methods

### Cell lines, reagents, and other materials

CCRF-CEM cells (human T-ALL cell line, ATCC CCL-119) carry a T-cell receptor *β*-chain rearrangement and harbor NOTCH1 mutations, a common genetic alteration in T-ALL associated with poor prognosis (21). Karyotyping analysis has shown this line to possess a near-diploid genome with chromosomal abnormalities including +8 and del(9p), which are frequently observed in high-risk T-ALL subsets. Moreover, it harbors mutations in several key genes relevant to ALL pathogenesis. For instance, in the p53 tumor suppressor gene, two heterozygous mutations have been identified at codons 175 and 248 in the DNA-binding domain (22). These mutations can potentially disrupt the normal function of p53, which is crucial for cell-cycle regulation, DNA repair, and apoptosis induction. Additionally, a frameshift mutation in the hypoxanthine-guanine phosphoribosyltransferase (HGPRT) gene has been reported in a sub-population of CCRF-CEM cells. Specifically, an Exon 8 deletion mutation in the HGPRT gene has been detected, which can affect purine salvage pathways and cellular metabolism (23). CCRF-CEM cells were obtained from Jiangsu Kaiji Biotechnology Co., Ltd. (Jiangsu, China), and DAC (A119533) was supplied by Aladdin (China). Pipettes were provided by Eppendorf (Germany). The Cell Counting Kit-8 (KGA9305-500), Annexin V-FITC/PI Apoptosis Detection Kit, and Cell Cycle Assay Kit (KGA1102-20) were purchased from Jiangsu Kaiji Biotechnology Co., Ltd. (Jiangsu, China). The cDNA First Strand Synthesis Kit was provided by TAKARA (RR820A; Shiga, Japan). The antibody panel comprised rabbit anti-GAPDH (KGC6102-1; Jiangsu Kaiji), rabbit anti-PI3K (ab191606; Abcam United Kingdom), rabbit anti-AKT (ab179463; Abcam UK), rabbit anti-mTOR (ab32028; Abcam United Kingdom), rabbit anti-PTEN (60300-1-Ig; Wuhan Sanying), rabbit anti-4EBP1 (60246-1-Ig; Wuhan Sanying), goat anti-rabbit IgG-HRP (KGC6202-0.1; Jiangsu Kaiji), and goat anti-mouse IgG-HRP (KGC6203-0.1; Jiangsu Kaiji), all from Jiangsu Kaiji Biotechnology Co., Ltd. (Jiangsu, China).

### Cell culture and drug therapy

Frozen CCRF-CEM cells were retrieved from liquid nitrogen and rapidly thawed in a 37°C water bath before being transferred to a centrifuge tube containing 4 mL of complete culture medium. Following centrifugation at 400 × *g* for 3 min, the cells were resuspended in 1 mL of medium, plated in culture flasks with additional medium, and incubated at 37°C under 5% CO_2_. Cells were treated with a range of DAC concentrations (100, 50, 10, 5, 1, 0.5, 0.1, 0.05, 0.025, 0.0125, and 0.00625 μM) for durations of 24, 48, 72, and 96 h to ascertain the optimal duration and concentration for activity. Cell viability and the effects of DAC were assessed using CCK8 assays, quantitative reverse transcription polymerase chain reaction (qRT-PCR), and flow cytometry.

### CCK8 assay to assess cell proliferation

DAC (molecular weight 228.21 g/mol) was dissolved in saline, stored at −20°C, and prepared in concentrations ranging from 0.00625–100 μM. A drug-free control group was established, with five replicates set for each concentration. The effect on CCRF-CEM cell proliferation was assessed after 24, 48, 72, and 96 h of treatment. Briefly, the CCK8 assay procedure involved collecting cells from each group, adjusting them to a density of 5 × 10^4^ cells/mL, and plating 100 μL per well in a 96-well plate, followed by incubation at 37°C under 5% CO_2_. At specific time points (24, 48, 72, and 96 h), 5 μL of CCK8 solution was added to each well, followed by a 2-h incubation and 10-min gentle shaking. Absorbance was then measured at 450 nm.

### Morphological and structural changes in apoptotic features

The cell line CCRF-CEM was treated with varying concentrations of DAC—0, 1.00 μmol/L, 10 μmol/L, and 100 μmol/L—for a duration of 48 h. Upon completion of the treatment, samples were retrieved from culture and subjected to embedding procedures, followed by trimming of the embedded blocks. Ultrathin sections (70 nm) were prepared using a Leica UC-7 ultramicrotome. These sections were then collected on copper grids and subjected to electron staining using lead as a staining agent. Finally, the stained sections were imaged using a transmission electron microscope (JEM1400, Japanese electronics) to observe and analyze the morphological and structural changes that occur during apoptosis (morada G3, Münster, Germany).

### Flow cytometry to assess cell apoptosis

Initially, cell counting was performed, and the cells were prepared to obtain a suspension with a density of 5 × 10^4^ cells/mL. Subsequently, 2 mL of this cell suspension was inoculated into each well of a six-well plate. Depending on the designated experimental groups, 0, 1.00 μmol/L, 10 μmol/L, and 100 μmol/L DAC solutions were added to the culture medium, alongside establishing a negative control group without any drug. All groups of cells were then cultured under these conditions for 72 h. Following this cultivation period, cells from each group were washed twice with PBS, and each wash was centrifuged at 1,000 rpm for 5 min to collect approximately 1 × 10^6^ cells. Harvested cells were resuspended in 500 μL of binding buffer. Sequentially, 5 μL of Annexin V-FITC was introduced to the cell suspension and thoroughly mixed. Immediately afterwards, 5 μL of propidium iodide was added, and the mixture was reincorporated thoroughly. The reaction proceeded in the dark at room temperature for 5–15 min to ensure accurate cell labeling. Finally, flow cytometry was employed to examine cells from all groups, facilitating the assessment and analysis of the apoptotic status in cells treated with varying concentrations of DAC.

### PI single staining method to assess cell cycle

After cell counting, a suspension was prepared at a density of 5 × 10^4^ cells/mL, and 2 mL of this suspension was added to each well of a six-well plate. Cells were treated with the respective drug concentrations or left untreated as a control. Following 72 h of drug treatment, cells were washed twice with PBS (1,000 rpm, 5 min), yielding 5 × 10^5^ cells. Cells were then fixed in 70% ethanol (KGF2203-100, Jiangsu Kaiji Biotechnology Co., Ltd) for 2 h at 4°C prior to staining, the fixative was removed by washing with PBS. Subsequently, 100 μL of RNase A was added, and cells were incubated at 37°C for 30 min. This was followed by the addition of 400 μL PI for staining after thorough mixing, with subsequent incubation in the dark at 4°C for another 30 min. Finally, red fluorescence emission at a 488 nm excitation wavelength was detected using flow cytometry, and the data were recorded.

### RT-PCR analysis

Cells were treated with DAC for 72 h prior to RNA extraction for RT-PCR analysis. Total RNA was isolated using TRIzol reagent (Invitrogen; TRIzol: KGF5101-100, Jiangsu Kaiji Biotechnology Co., Ltd) following the manufacturer’s instructions. Briefly, cells were lysed in 1 mL TRIzol, mixed with 200 μL chloroform by vortexing for 15 s, and incubated at room temperature for 3 min. After centrifugation at 12,000 × g for 15 min at 4°C, the aqueous phase was collected. RNA was precipitated by adding an equal volume of isopropanol, followed by centrifugation at 12,000 × g for 10 min at 4°C. The resulting RNA pellet was washed with 75% ethanol, air-dried, and dissolved in RNase-free water. RNA concentration and purity (OD_260_/_280_ ratio) were determined using a NanoDrop 2000 spectrophotometer. Potential genomic DNA contamination was assessed by PCR amplification without reverse transcriptase (RT-minus control). Reverse transcription was performed to synthesize cDNA. Quantitative RT-PCR was carried out using the prepared cDNA, specific primers, TaqMan probes, and PCR reagents (TaKaRa RR036B, Japan) in a reaction system that dynamically monitored accumulating fluorescence signals over multiple temperature cycles. Genes analyzed for their roles in apoptosis regulation included pro-survival factors (*PI3K*, *AKT*, *mTOR*), the anti-survival factor *PTEN*, and mTOR effectors (*P70S6*, *4EBP1*). miRNAs (e.g., miR-92b-3p, miR-193a-3p, miR-19, miR-21, miR-22, miR-93a, miR-193a, miR-221, miR-223, miR-20) were selected based on prior reports linking them to apoptosis in ALL. GAPDH and U6 snRNA served as endogenous controls for mRNA and miRNA quantification, respectively. Amplification efficiency for each primer/probe set (92–105%) was confirmed by standard curve analysis using 10-fold serial dilutions of cDNA. Relative gene expression levels were calculated using the 2^(−ΔΔCt)^ method. All primers and probes (sequences provided in Table 1) were designed using Primer3 software and synthesized by Sangon Biotech (Shanghai, China).

**Table 1:** RT-PCR primers used in this study.

Gene	Forward Primer (5′ → 3′)	Reverse Primer (5′ → 3′)	Amplicon length (bp)
PI3K	CGAGTGGTTGGGCAATGAAA	CTCGCAACAGGTTTTCAGCT	134
AKT	TACTACGCCATGAAGATCCTCAA	CGTACTCCATGACAAAGCAGAG	166
PTEN	ACCCACCACAGCTAGAACTT	AGTTCGTCCCTTTCCAGCTT	113
4EBP1	GTGTCGGAACTCACCTGTG	CCGCTTATCTTCTGGGCTATTG	136
mTOR	GTCAGAATCCAAGTCAAGTCAGG	ATGGTGTGATGATGAGAGAGTGA	159
P70S6	ACCTCACACAAGAAGCCAGA	GAGCTTGAACTTCTCCAGCG	102
GAPDH	CAAATTCCATGGCACCGTCA	AGCATCGCCCCACTTGATTT	109
hsa-miR-19a-3p	ACACTCCAGCTGGGTGTGCAAATCTATGCAA	- (stem-loop RT primer)	71
hsa-miR-20a-3p	ACACTCCAGCTGGGACTGCATTATGAGCAC	- (stem-loop RT primer)	65
hsa-miR-21–3p	ACACTCCAGCTGGGCAACACCAGTCGATG	- (stem-loop RT primer)	66
hsa-miR-22-3p	ACACTCCAGCTGGGAAGCTGCCAGTTGAAG	- (stem-loop RT primer)	74
hsa-miR-92b-3p	ACACTCCAGCTGGGTATTGCACTCGTCCCG	- (stem-loop RT primer)	82
hsa-miR-93-3p	ACACTCCAGCTGGGACTGCTGAGCTAGCAC	- (stem-loop RT primer)	71
hsa-miR-193a-3p	ACACTCCAGCTGGGAACTGGCCTACAAAGT	- (stem-loop RT primer)	76
hsa-miR-221–3p	ACACTCCAGCTGGGAGCTACATTGTCTGCTG	- (stem-loop RT primer)	87
hsa-miR-223-3p	ACACTCCAGCTGGGTGTCAGTTTGTCAAAT	- (stem-loop RT primer)	89
U6	CTCGCTTCGGCAGCACA	AACGCTTCACGAATTTGCGT	-

### Western blotting

Cells were treated with DAC for 72 h, then lysed to extract total proteins. Western blotting was performed in triplicate (biological replicates) using independent cell cultures. After extracting the total cellular proteins, protein concentrations were determined using a BCA kit. Each group loaded 20 μg of protein onto a system with 12% resolving gel and 5% stacking gel. Electrophoresis was initially performed at 80 V for 40 min to concentrate proteins, followed by 120 V for 50 min for separation. Subsequently, proteins were transferred to a membrane at a constant voltage of 90 V for 1 h and blocked with 5% non-fat milk at room temperature (or overnight at 4°C). Primary antibodies (Bad 1:1,000, Bcl-2 1:1,000, cleaved-caspase-3 1:1,000, and GAPDH 1:1,000) were applied to the membrane and incubated at room temperature for 1 h. Following membrane washing, the secondary antibody goat anti-rabbit IgG (1:10,000) was applied and incubated at room temperature for another hour. Thereafter, ECL reagent was used for chemiluminescence development, and images were captured using the Bio-Rad ChemiDoc Touch imaging system. Gel-Pro32 software was employed for the grayscale analysis of the results, and each experiment was repeated three times to ensure reliability.

### *In vivo* studies

A total of 21 five-week-old female BALB/c nude mice were obtained from SPF Biotechnology Co., Ltd. (Suzhou, China). Following a one-week acclimation period, we harvested cultured human acute lymphoblastic T-lymphocyte CCRF-CEM cells, mixed them with high-concentration matrix gel and PBS at a 1:1 ratio, adjusted the cell concentration to 1 × 10^8^ cells/mL, and injected 0.1 mL of this suspension into the right axillary subcutaneous tissue of each mouse to establish a tumor xenograft model. The diameter of the transplanted tumors was monitored using a Vernier caliper. Once they reached 100 mm^3^ in size, the mice were randomly divided into three groups. The control group received daily intraperitoneal injections of 0.2 mL saline, the DAC treatment group was administered 20 mg/kg DAC daily via intraperitoneal injection, and the doxorubicin group was administered 3 mg/kg doxorubicin intraperitoneally on days 1, 6, and 12. Every 3 days, the weights of the nude mice were recorded, and the long diameter (A) and short diameter (B) of the tumors were measured to calculate the tumor volume. Additionally, the overall health status of the mice, including skin color and activity levels, was observed and documented. Mice were euthanized using carbon dioxide (CO_₂_) as per American Veterinary Medical Association guidelines: animals were exposed to 70% CO_₂_ in air (flow rate 20% chamber volume/min), followed by 100% CO_₂_ until loss of righting reflex and absence of vital signs (no respiration, corneal reflex, or paw pinch response). Euthanasia was confirmed by 2 additional minutes of 100% CO_₂_ exposure.

### Statistical analysis

Descriptive statistics were presented as the mean ± standard deviation (mean ± SD), while multiple-group comparisons were conducted using one-way analysis of variance (ANOVA). Subsequent explorations of post-ANOVA differences were performed using the least significant difference test. All hypothesis tests were two-tailed, with statistical significance set at *p* < 0.05. The entire data analysis process was performed using SPSS software (version 26.0).

## Results

### Selection of optimal concentration and time of action of DAC

**Figure 1:**
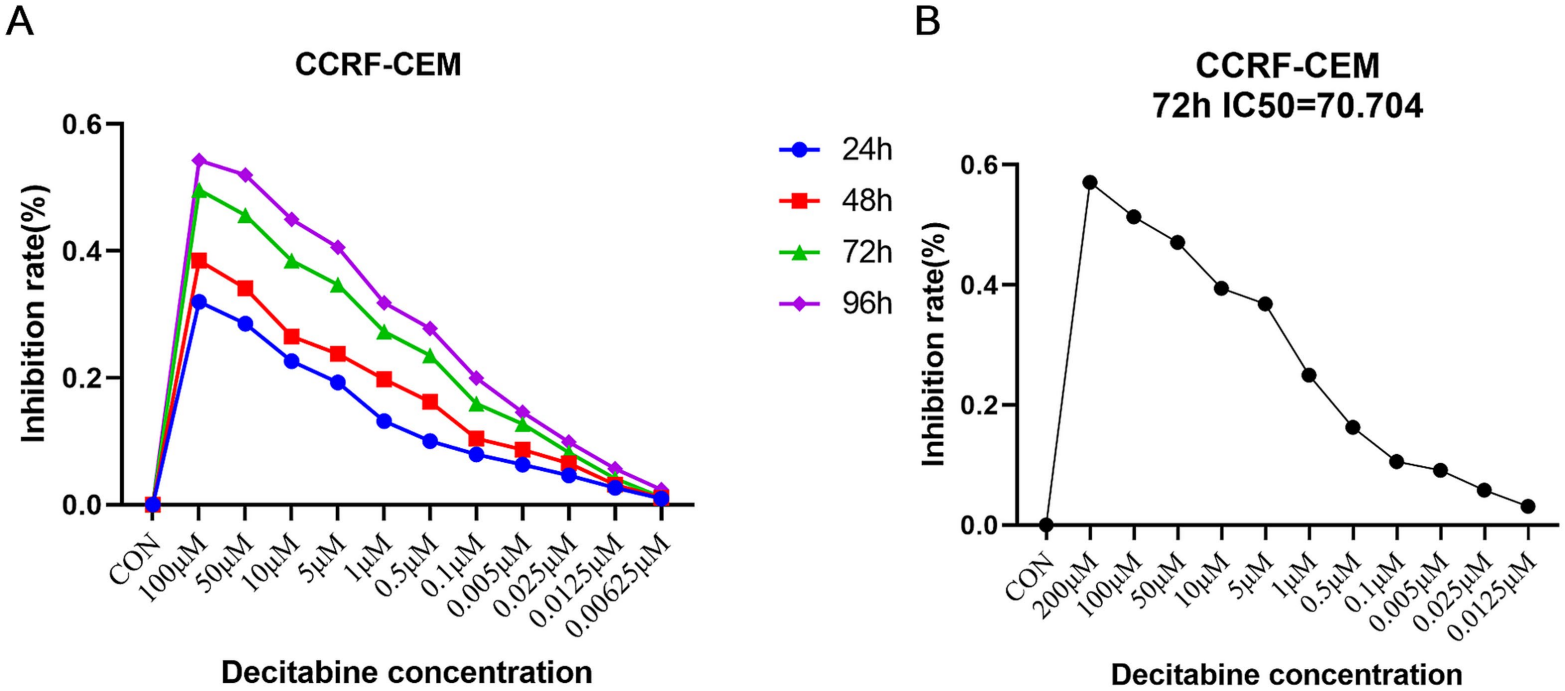
Selection of the optimal concentration and time of action of DAC. **(A)** Detection of inhibition rate in each group; **(B)** The threshold concentration of DAC required to achieve 50% inhibition of cell growth after 72 h. **(A)** CCK-8 assay showing dose- and time-dependent inhibition of CCRF-CEM cells by DAC. **(B)** IC_50_ determination after 72 h of treatment. Data are mean ± standard deviation (SD) of three independent biological replicates (*n* = 3).

CCRF-CEM cells were treated with various concentrations of DAC for 24, 48, 72, and 96 h. To assess the proliferative activity of the cells in each group, a CCK8 assay was employed. The results demonstrated that as the exposure time to DAC increased along with its concentration, the inhibitory effect on cell growth increased, manifesting a consistently increasing inhibition rate (Figure 1A). Notably, after 72 h of treatment, the IC_50_ of DAC after 72 h was 70.704 μM, as illustrated in Figure 1B.

### Morphological and structural changes

**Figure 2:**
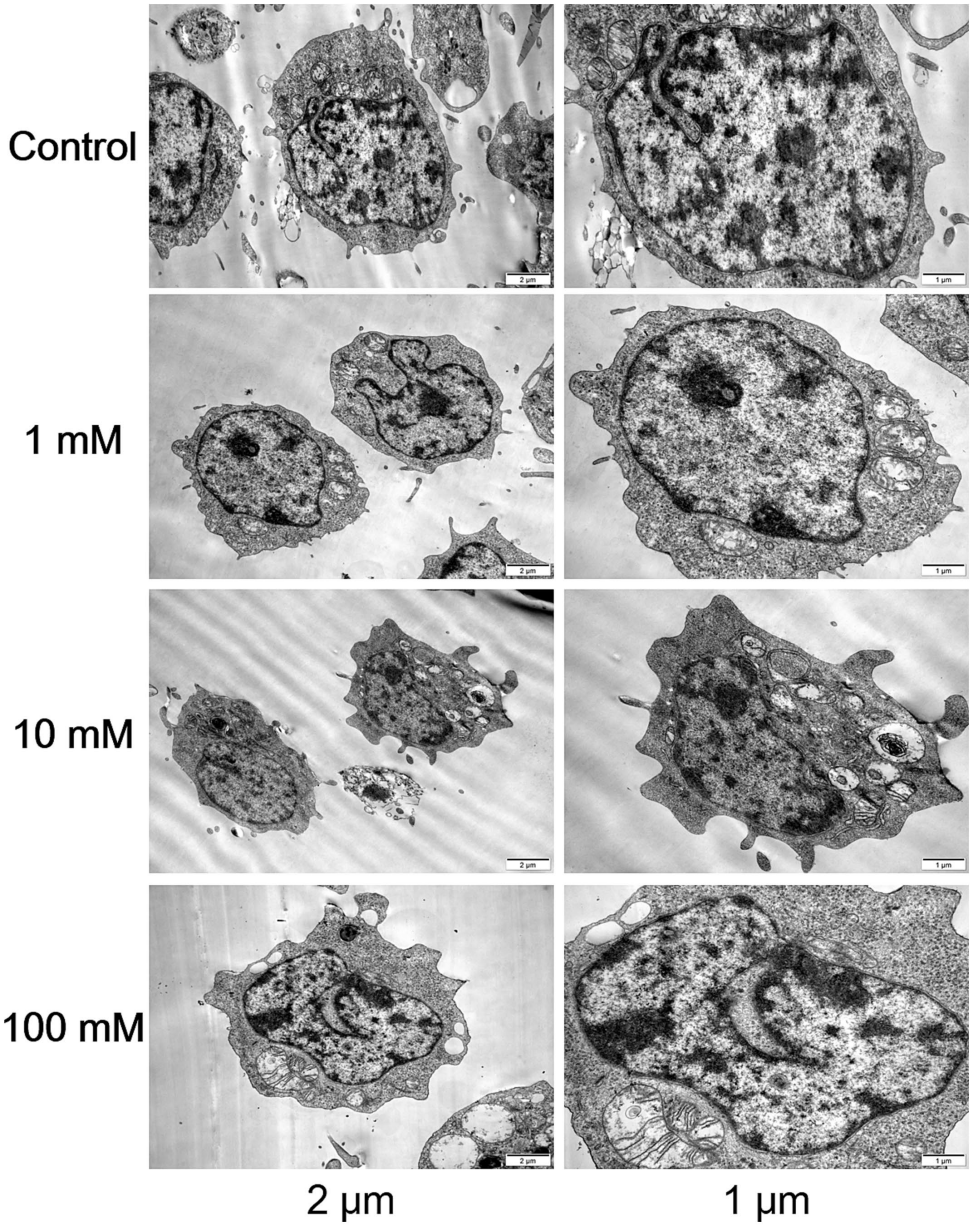
Morphological and structural changes in each group. Transmission electron microscopy images of CCRF-CEM cells treated with DAC (0, 1, 10, 100 μM) for 48 h, showing nuclear condensation and apoptotic bodies. Images are representative of three independent experiments.

Following DAC treatment in CCRF-CEM cells, high doses resulted in more pronounced morphological and structural changes that induced apoptosis. Cells treated with the high dose displayed heightened nuclear condensation, fragmentation, and profusion of apoptotic bodies, accompanied by vesicular protrusions on the cellular membrane, which are characteristic features of late-stage apoptosis. In contrast, while low-dose DAC also initiated apoptosis, these changes were relatively milder, with a lower proportion of apoptotic cells and less evident apoptotic traits compared to the high-dose treatment. This suggested that DAC dosage had a direct impact on the intensity and kinetics of apoptosis induction (Figure 2).

### Cell apoptosis and cell cycle

**Figure 3:**
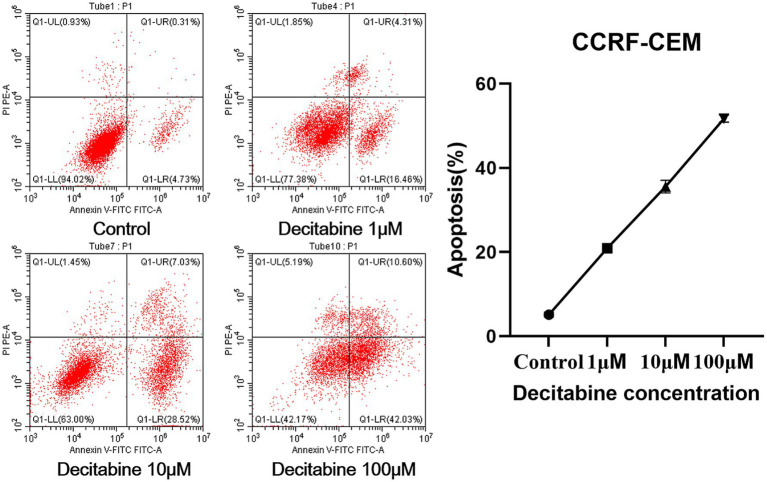
The apoptotic rate in each group. Annexin V-FITC/PI staining results after 72 h of DAC treatment. Apoptotic rates were analyzed by flow cytometry. Data are mean ± SD of three biological replicates (*n* = 3).

**Figure 4:**
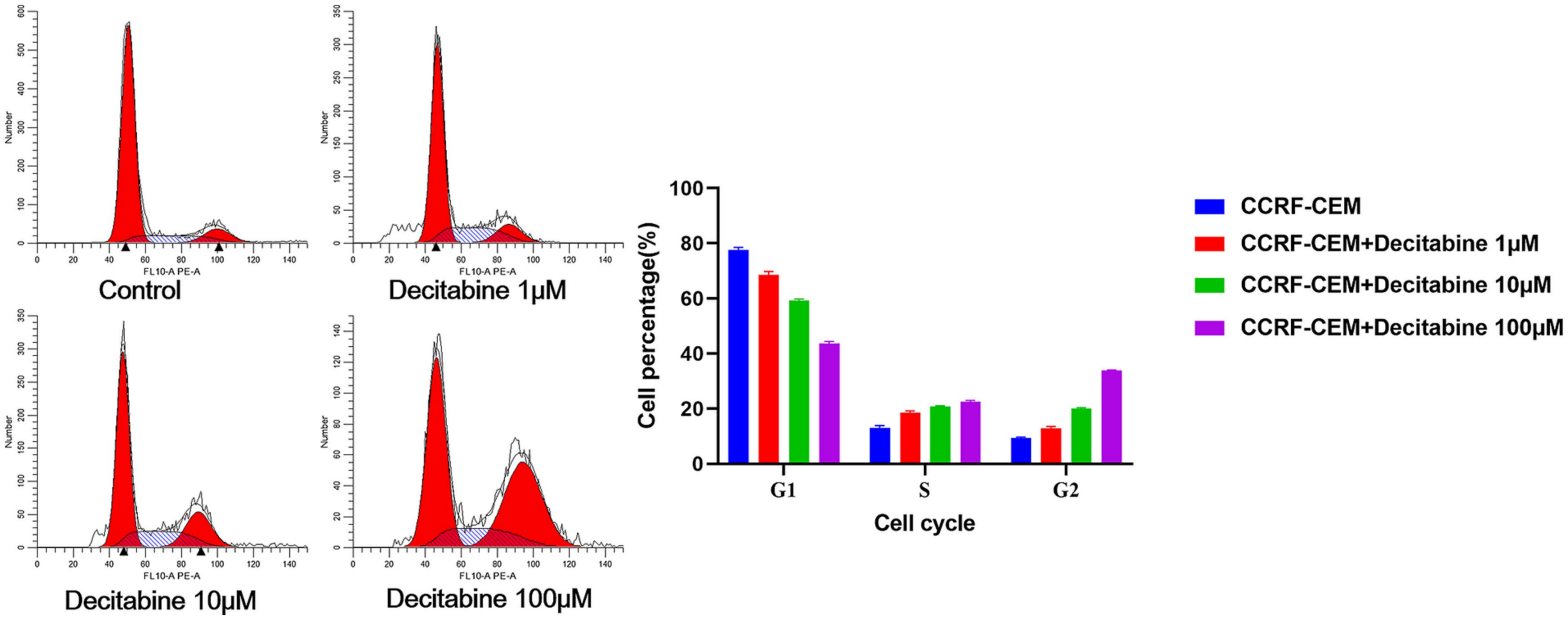
The cell percentage after DAC treatments in the G1, S, and G2 phases. Cell cycle distribution analyzed by PI staining after 72 h of DAC treatment. Data represent mean ± SD of three biological replicates (*n* = 3). CCRF-CEM vs. CCRF-CEM + Decitabine 1 μM, *p* < 0.05; CCRF-CEM + Decitabine 1 μM vs. CCRF-CEM + Decitabine 10 μM, *p* < 0.05; CCRF-CEM + Decitabine 10 μM vs. CCRF-CEM + Decitabine 100 μM, *p* < 0.05.

The Annexin V-FITC/PI double staining method, when applied through flow cytometry for apoptosis detection, revealed a significant increase in the apoptotic rate as the DAC concentration increased (Figure 3). Our findings further indicated that with an increase in DAC dose, there was a notable decrease in the number of cells in the G1 phase, whereas in the S and G2 phases, a significant increase in cell count was observed as the DAC concentration increased (Figure 4).

### Expression levels of apoptosis-related genes and proteins

**Figure 5:**
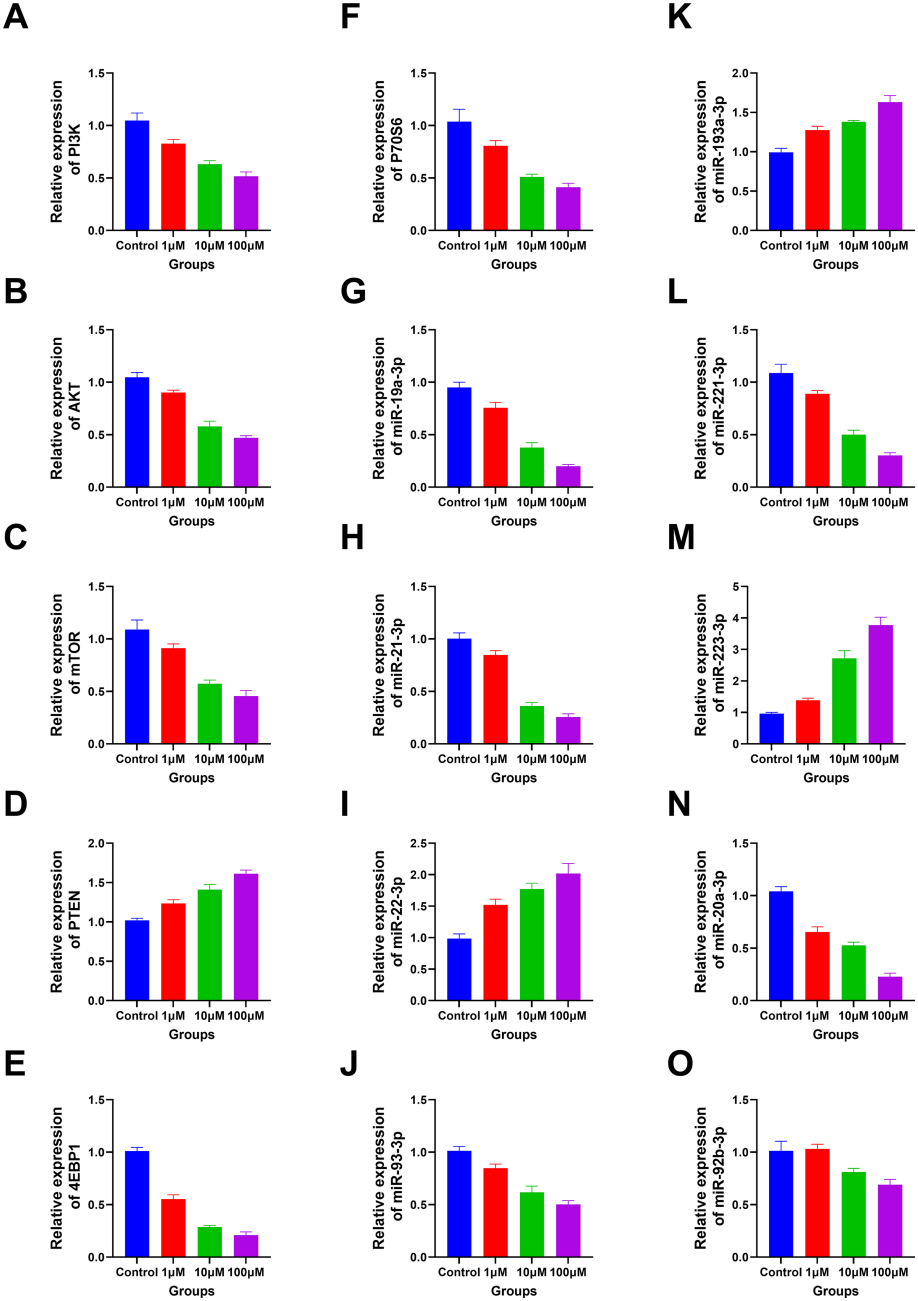
The apoptosis-related genes expression in each group. **(A)** PI3K; **(B)** AKT; **(C)** mTOR; **(D)** PTEN; **(E)** 4EBP1; **(F)** P70S6; **(G)** miR-19a-3p; **(H)** miR-21–3p; **(I)** miR-22-3p; **(J)** miR-93-3p; **(K)** miR-193a-3p; **(L)** miR-221–3p; **(M)** miR-223-3p; **(N)** miR-20a-3p; **(O)** miR-92b-3p. **(A–N)** Decitabine 1 μM vs. control, *p* < 0.05; Decitabine 10 μM vs. Decitabine 1 μM, *p* < 0.05; Decitabine 100 μM vs. Decitabine 10 μM, *p* < 0.05. **(O)** Decitabine 10 μM vs. Decitabine 1 μM, *p* < 0.05; Decitabine 100 μM vs. Decitabine 10 μM, *p* < 0.05. RT-PCR analysis of apoptosis-related genes after 72 h of DAC treatment. Data are mean ± SD of three biological replicates (*n* = 3), normalized to GAPDH or U6.

**Figure 6:**
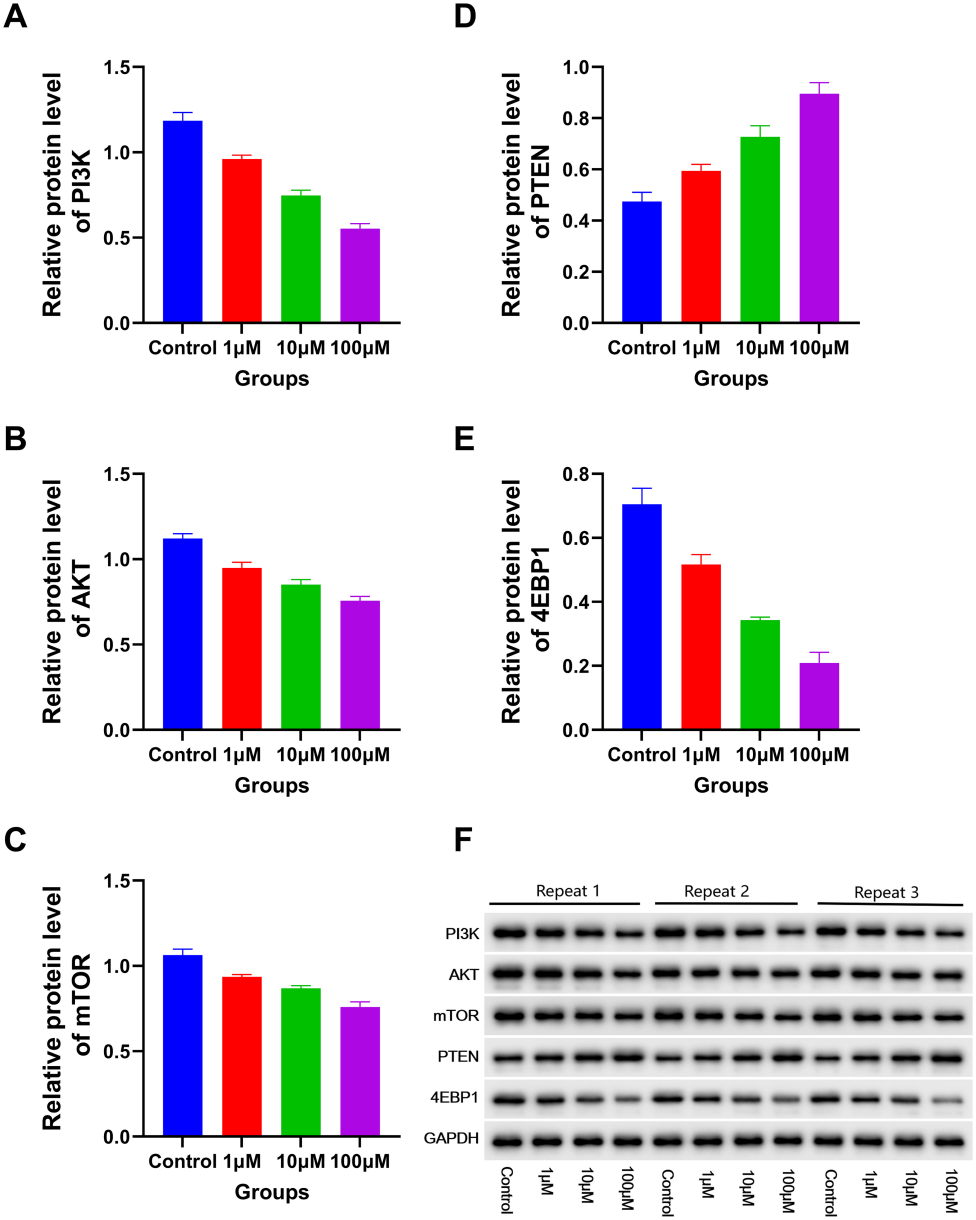
The expression levels of apoptosis-related proteins in each group. Western blotting analysis of apoptotic proteins (72 h post-DAC treatment). Band intensities were quantified and normalized to GAPDH. Data are mean ± SD of three biological replicates (*n* = 3). **(A)** PI3K; **(B)** AKT; **(C)** mTOR; **(D)** PTEN; **(E)** 4EBP1. Decitabine 1 μM vs. control, *p* < 0.05; Decitabine 10 μM vs. Decitabine 1 μM, *p* < 0.05; Decitabine 100 μM vs. Decitabine 10 μM, *p* < 0.05. **(F)** WB analysis for the expression levels of apoptosis-related proteins in each group.

The RT-PCR results revealed a significant decline in the expression levels of *PI3K, P70S6, AKT, miR-19a-3p, miR-221–3p, mTOR, miR-21–3p, miR-20a-3p, 4EBP1, miR-93-3p*, and *miR-92b-3p* with increasing DAC dosage. Conversely, a marked elevation in the expression levels of *miR-193a-3p, miR-223-3p, PTEN*, and *miR-22-3p* was observed under the same conditions (Figure 5). In addition, the protein expression levels of PI3K, AKT, 4EBP1, and mTOR gradually decreased as DAC dosage increased, whereas the expression level of PTEN increased with increasing DAC concentration (Figure 6).

### *In vivo* studies

**Figure 7:**
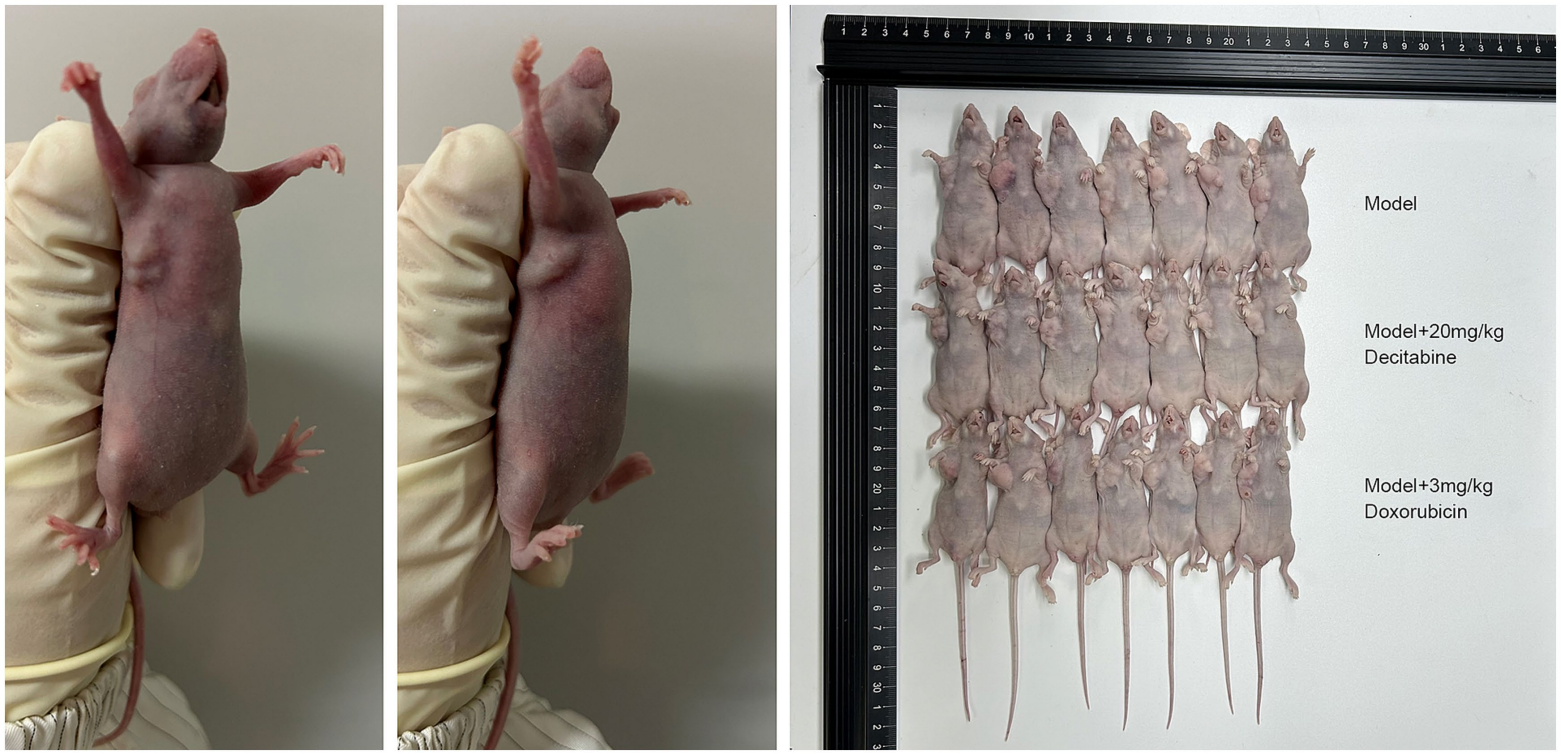
The subcutaneous tumor formation in each group. Representative images of subcutaneous tumors in nude mice (40 days post-implantation). Mice were treated with saline, DAC (20 mg/kg), or doxorubicin (3 mg/kg). Images are from *n* = 7 mice per group.

**Figure 8:**
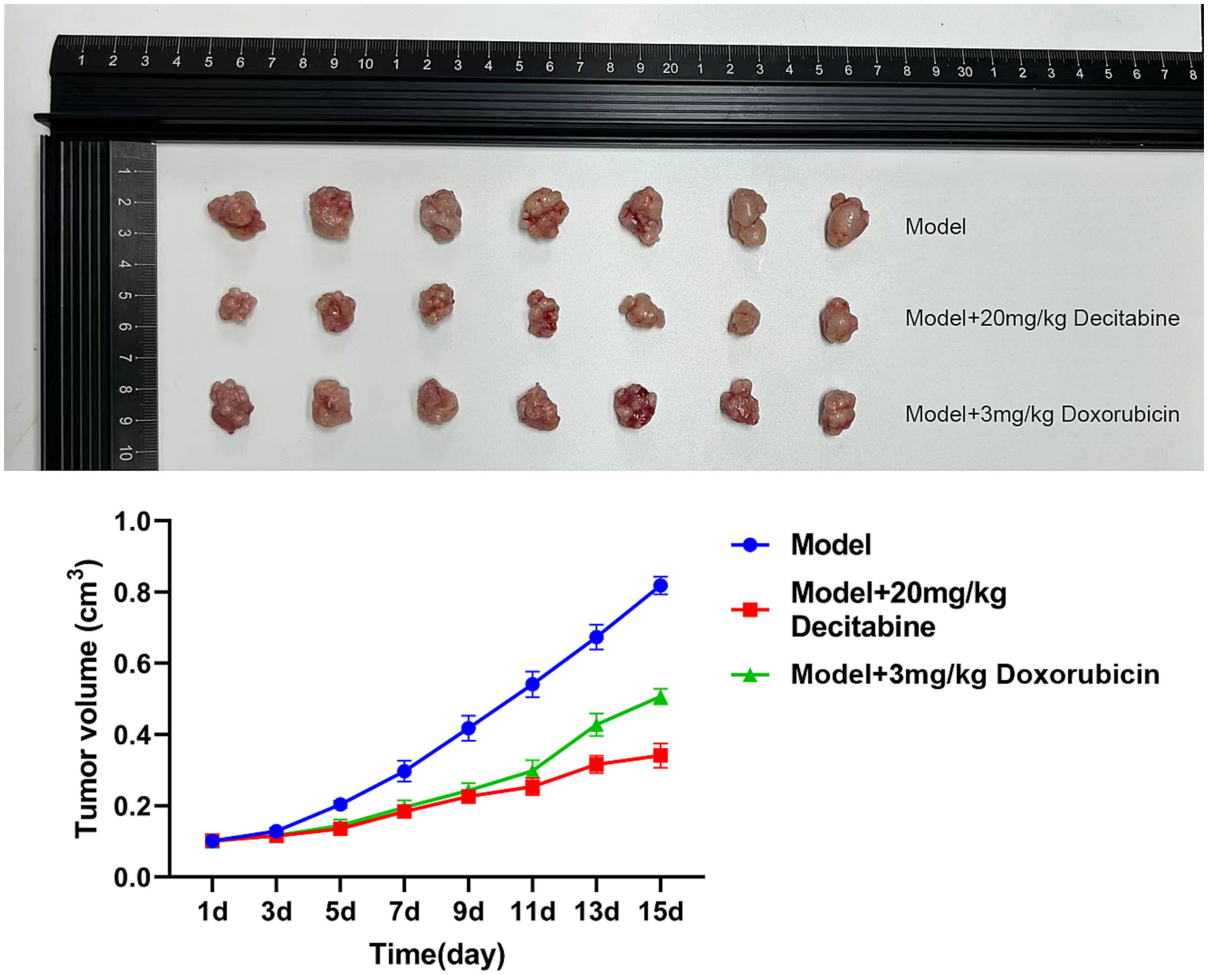
The inhibitory effect of DAC on tumor volume. Tumor volume kinetics in nude mice (mean ± SD, *n* = 7 per group). DAC significantly reduced tumor growth compared to doxorubicin and control (*p* < 0.01). Tumor volume was calculated as V = 0.5 × A × B^2^.

CCRF-CEM cells were implanted into the subcutaneous tissue of nude mice. After 40 days of cell cultivation, tumors were formed with a size of approximately 100 mm^3^. Detailed information on the subcutaneous tumor formation is shown in Figure 7. Over time, tumor volume in the model group gradually increased. In contrast, the doxorubicin-treated group exhibited delayed tumor progression, whereas the DAC-treated group demonstrated a more pronounced inhibitory effect, surpassing that of the doxorubicin group (Figure 8).

## Discussion

This study delved into the mechanisms by which DAC operates in treating ALL, revealing that high-dose DAC demonstrates significantly superior efficacy compared with low-dose DAC in inhibiting tumor cell proliferation and promoting tumor cell apoptosis. Further animal experiments corroborated that DAC can potently suppress tumor growth, with its tumor inhibition efficiency markedly outperforming that of the conventional drug doxorubicin. Moreover, our study revealed that DAC substantially reduced the expression levels of *PI3K, P70S6, AKT, miR-19a-3p, miR-221–3p, mTOR, miR-21–3p, miR-20a-3p, 4EBP1, miR-93-3p*, and *miR-92b-3p*, and this suppressive effect was augmented as the DAC dosage increased. Conversely, DAC facilitated the expression of *miR-193a-3p, miR-223-3p, PTEN*, and *miR-22-3p*, and its capacity to induce expression significantly increased as the dose increased. Notably, CCRF-CEM cells exhibit molecular features of the ‘early T-cell precursor’ subtype, including CD1a−/CD1b−/CD8− immunophenotype and NOTCH1 mutations, which represent 15–20% of T-ALL cases with inferior outcomes (24). The observed del(9p) mirrors cytogenetic abnormalities in high-risk T-ALL, further validating this model’s relevance to aggressive disease subsets. Thus, our findings specifically inform T-ALL therapy, warranting validation in B-ALL models.

While previous studies have confirmed that DAC can significantly enhance the treatment outcomes of ALL (25–29), a comprehensive understanding of its mechanisms of action remains to be achieved. DAC notably inhibited the proliferation of CCRF-CEM cells and induced apoptosis, with the effect intensifying as the dosage increased (30). It exerts diverse effects at various stages of the cell cycle, suppressing DNA methyltransferases to facilitate DNA demethylation, particularly in abnormally methylated genomic regions such as those in tumor suppressor gene promoters. This reactivates silenced tumor suppressor genes and restores normal cell growth and apoptotic pathways, thereby regulating cell proliferation and apoptosis (31). As a cytosine analog, erroneous incorporation of DAC into DNA, particularly during replication, can impede DNA synthesis and damage DNA. If left unrepaired, the damage accumulates and ultimately induces apoptosis (32). Higher doses are more effective at blocking S-phase DNA replication and vigorously suppressing proliferation. Lower doses may interfere with G1/S or G2/M transitions, disrupting cell cycle progression, albeit with a milder apoptotic induction effect (33).

In previous investigation employing the MOLT-4 T-ALL cell line (34), the IC_50_ value for DAC was determined to be 50 μM. In contrast, the present study using the CCRF-CEM cell line revealed an IC_50_ of 70.7 μM. This variance may be attributed to the distinct molecular characteristics of these cell lines. For instance, MOLT-4 cells display a defective p21(WAF1) induction mechanism following DNA damage despite normal p53 function. This defect is associated with epigenetic repression mechanisms, including reduced acetylation of H3K9 in the promoter region and increased CpG methylation in the first exon/intron. In CCRF-CEM cells, the basal levels of key signaling molecules and epigenetic regulators might be different, leading to a higher resistance to DAC. Another contributing factor to the difference in DAC sensitivity could be the activation of survival pathways. T-ALL cell lines are known to rely on different survival signaling pathways, and the CCRF-CEM cells may have a more robust activation of pathways that counteract the effects of decitabine (35). Additionally, the MOLT-4 cells, with their specific genetic and epigenetic landscape, may be more permissive to the demethylating and anti-leukemic effects of DAC. Understanding these differences can provide a more comprehensive view of how DAC exerts its effects in T-ALL and may inform the development of more targeted treatment strategies.

In the present study, the expression levels of *PI3K, P70S6, AKT, miR-19a-3p, miR-221–3p, mTOR, miR-21–3p, miR-20a-3p, 4EBP1, miR-93-3p*, and *miR-92b-3p* were significantly reduced in the DAC group, whereas those of *miR-193a-3p, miR-223-3p, PTEN*, and *miR-22-3p* were increased. Moreover, DAC significantly reduced PI3K, AKT, 4EBP1, and mTOR protein expression levels, whereas PTEN protein levels were significantly increased. Previous studies have demonstrated that DAC inhibits the PI3K/AKT/mTOR pathway through PTEN, partially reducing the viability of MOLT4 cells, particularly at low concentrations, which is consistent with the findings of the present study (18). However, our study revealed that DAC significantly suppresses the expression level of 4EBP1. A marked reduction in 4EBP1 expression is a pivotal consequence of DAC intervention (36, 37). 4EBP1 modulates the function of eIF4E (eukaryotic initiation factor 4E), which is crucial for initiating protein synthesis. When 4EBP1 expression is inhibited, eIF4E is no longer effectively restrained, leading to its overactivation, which paradoxically does not directly stimulate protein synthesis but instead triggers stress responses and apoptotic pathways in various cell types (38). Consequently, the disruption of protein synthesis due to 4EBP1 repression, along with subsequent stress reactions, markedly impaired the proliferative capacity of CCRF-CEM cells and initiated apoptosis. This cascade of events demonstrates how DAC effectively controls malignant cell proliferation and promotes apoptosis by precisely targeting specific nodes within the epigenetic regulatory network.

A notable limitation of this study is the reliance on a single T-ALL cell line (CCRF-CEM), which may not fully capture the genetic heterogeneity of clinical T-ALL. Future studies should leverage publicly available RNAseq datasets to analyze the expression dynamics of *PI3K, AKT, 4EBP1, mTOR,* and *PTEN* in primary ALL patient samples treated with decitabine. Such analyses could identify these genes as potential biomarkers for therapeutic response, particularly by comparing expression changes in decitabine-sensitive vs. resistant cases.

## Conclusion

This study uncovered DAC’s efficacy in inhibiting the proliferation of CCRF-CEM cells, accelerating apoptosis, and suppressing tumor growth, with its effectiveness increasing as the dose increased. Furthermore, we conclusively demonstrated that DAC significantly downregulated the expression of PI3K, AKT, 4EBP1, and mTOR proteins, while concurrently boosting the levels of PTEN protein; these regulatory effects became more pronounced with increasing DAC dosage. Validation of these findings in primary ALL patient data and exploration of PI3K/PTEN/4EBP1 as therapeutic response markers represent important future directions. These findings suggest DAC may be a promising agent for NOTCH1-mutated T-ALL subsets, warranting validation in diverse preclinical models.

## Data Availability

The original contributions presented in the study are included in the article/Supplementary material, further inquiries can be directed to the corresponding author.
