# Supplementary material for: Mechanism of action of decitabine in treating acute lymphoblastic leukemia
Source: Front Med (Lausanne). 2025 Jul 30;12:1614592. doi: 10.3389/fmed.2025.1614592 (PMC12343678; doi:10.3389/fmed.2025.1614592)
Supplement: Supplementary file 1 [file Table_1.DOCX]

| 4EBP1  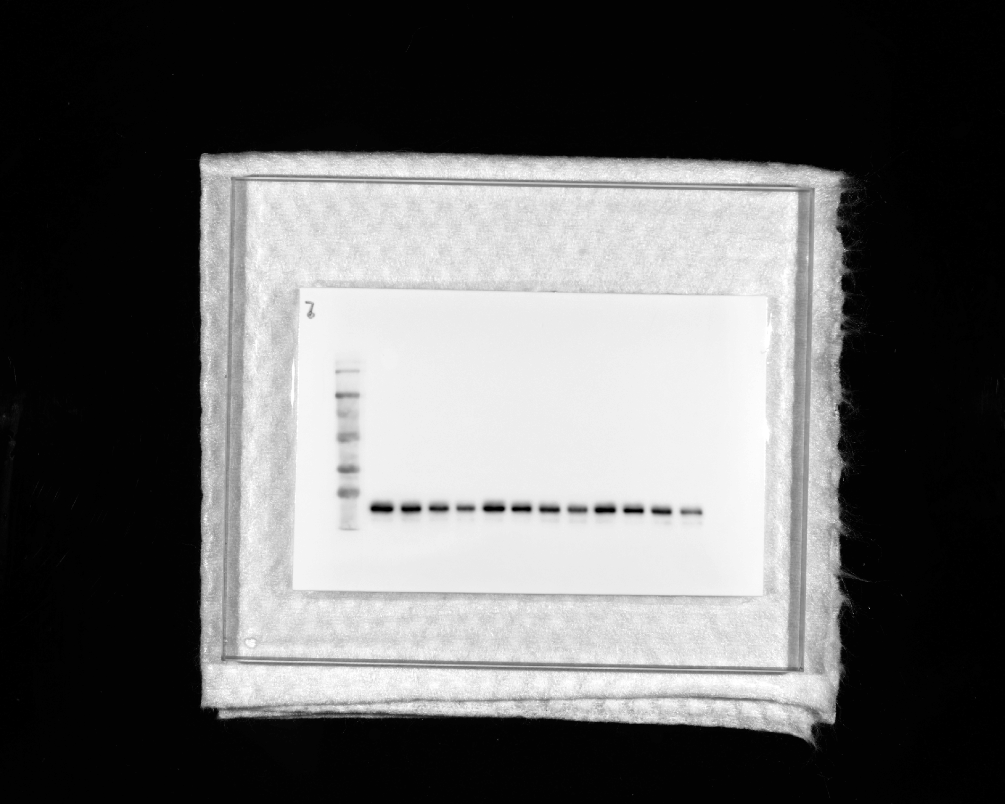  120 kDa  100 kDa  70 kDa  50 kDa  40 kDa  30 kDa  25 kDa  14 kDa  CCRF-CEM  CCRF-CEM+1μM地西他滨  CCRF-CEM+10μM地西他滨  CCRF-CEM+100μM地西他滨  CCRF-CEM  CCRF-CEM+1μM地西他滨  CCRF-CEM+10μM地西他滨  CCRF-CEM+100μM地西他滨  CCRF-CEM  CCRF-CEM+1μM地西他滨  CCRF-CEM+10μM地西他滨  CCRF-CEM+100μM地西他滨 |
| --- |
| AKT  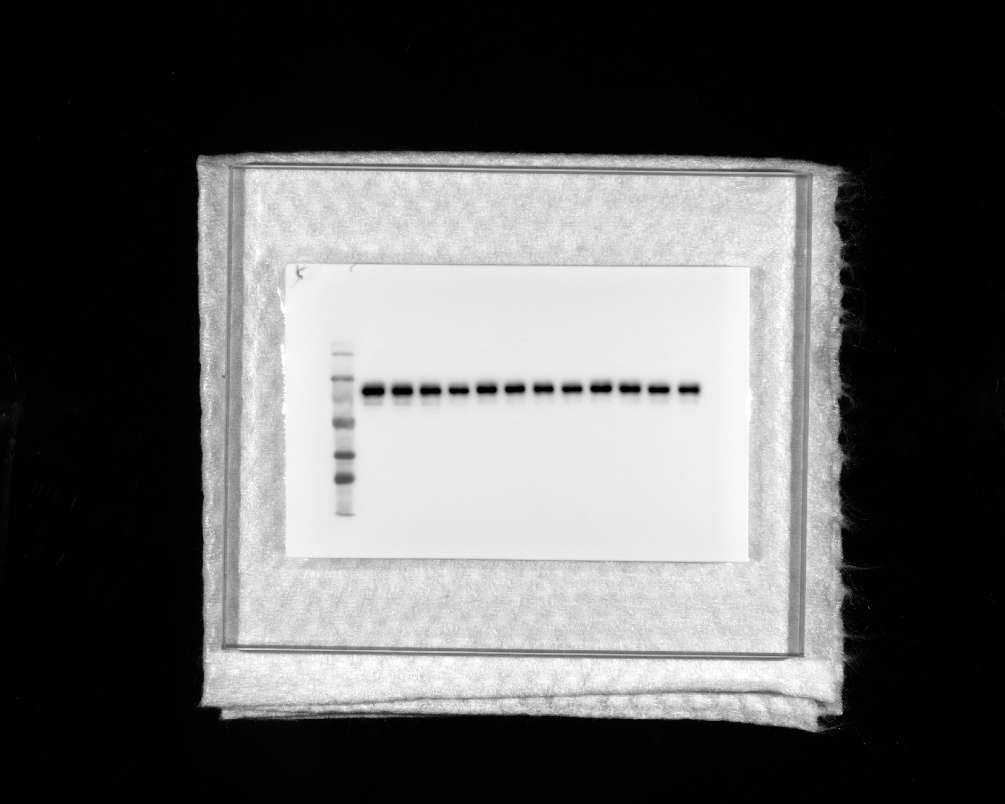  120 kDa  100 kDa  70 kDa  50 kDa  40 kDa  30 kDa  25 kDa  14 kDa  CCRF-CEM+100μM地西他滨  CCRF-CEM+10μM地西他滨  CCRF-CEM+1μM地西他滨  CCRF-CEM  CCRF-CEM+1μM地西他滨  CCRF-CEM+10μM地西他滨  CCRF-CEM+100μM地西他滨  CCRF-CEM  CCRF-CEM+1μM地西他滨  CCRF-CEM+10μM地西他滨  CCRF-CEM+100μM地西他滨  CCRF-CEM |
| GAPDH  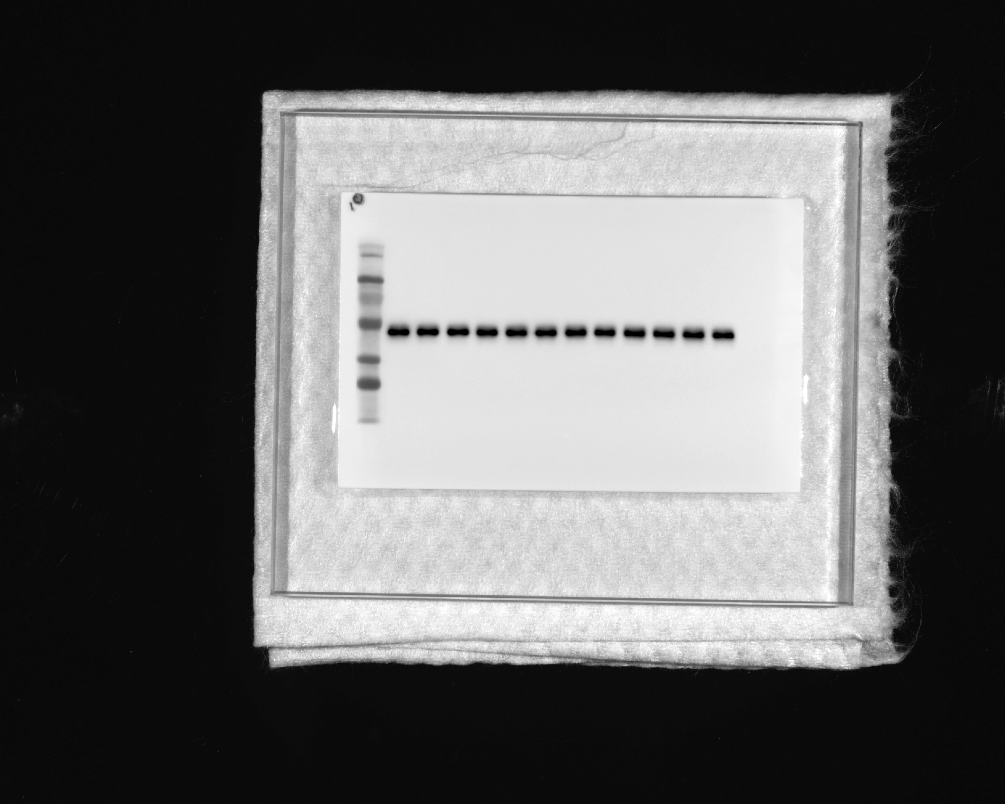  CCRF-CEM+100μM地西他滨  CCRF-CEM+10μM地西他滨  CCRF-CEM+100μM地西他滨  CCRF-CEM  CCRF-CEM+1μM地西他滨  CCRF-CEM+10μM地西他滨  CCRF-CEM+100μM地西他滨  CCRF-CEM  CCRF-CEM+1μM地西他滨  CCRF-CEM+10μM地西他滨  CCRF-CEM  CCRF-CEM+1μM地西他滨  120 kDa  100 kDa  70 kDa  50 kDa  40 kDa  30 kDa  25 kDa  14 kDa |
| m-TOR  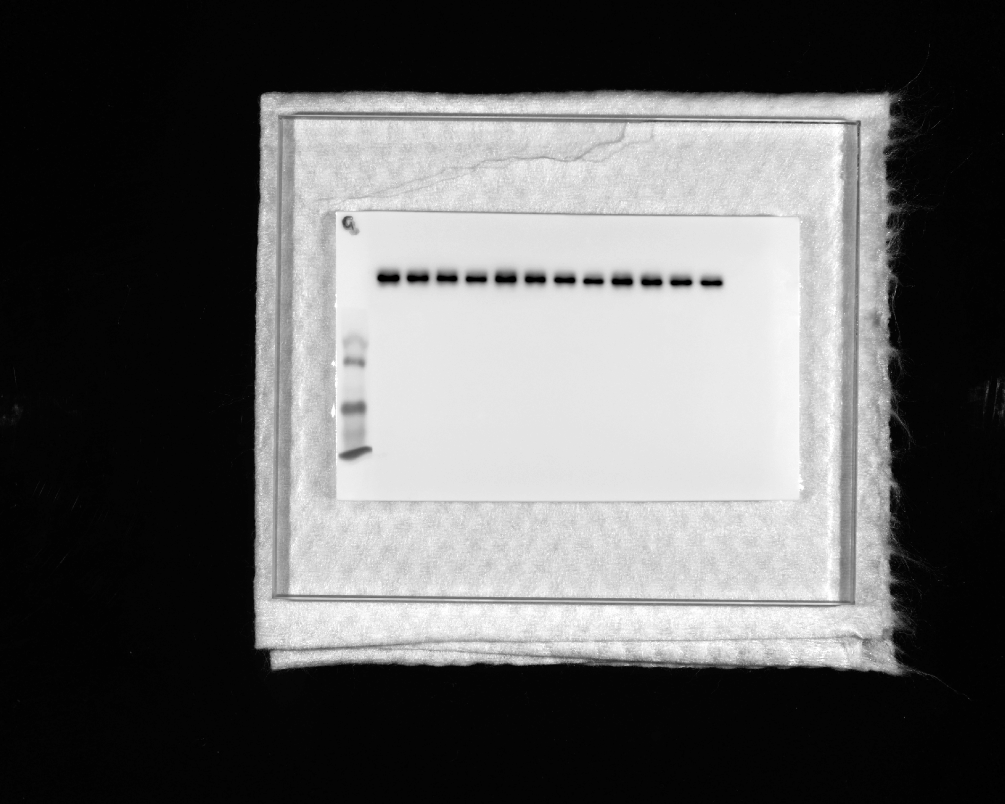  100 kDa  70 kDa  50 kDa  120 kDa  CCRF-CEM  CCRF-CEM+1μM地西他滨  CCRF-CEM+10μM地西他滨  CCRF-CEM+100μM地西他滨  CCRF-CEM  CCRF-CEM+1μM地西他滨  CCRF-CEM+10μM地西他滨  CCRF-CEM+100μM地西他滨  CCRF-CEM  CCRF-CEM+1μM地西他滨  CCRF-CEM+10μM地西他滨  CCRF-CEM+100μM地西他滨 |
| PI3K  CCRF-CEM  CCRF-CEM+1μM地西他滨  CCRF-CEM+10μM地西他滨  CCRF-CEM+100μM地西他滨  CCRF-CEM  CCRF-CEM+1μM地西他滨  CCRF-CEM+10μM地西他滨  CCRF-CEM+100μM地西他滨  CCRF-CEM  CCRF-CEM+1μM地西他滨  CCRF-CEM+10μM地西他滨  CCRF-CEM+100μM地西他滨  120 kDa  100 kDa  70 kDa  50 kDa  40 kDa  30 kDa  25 kDa  14 kDa  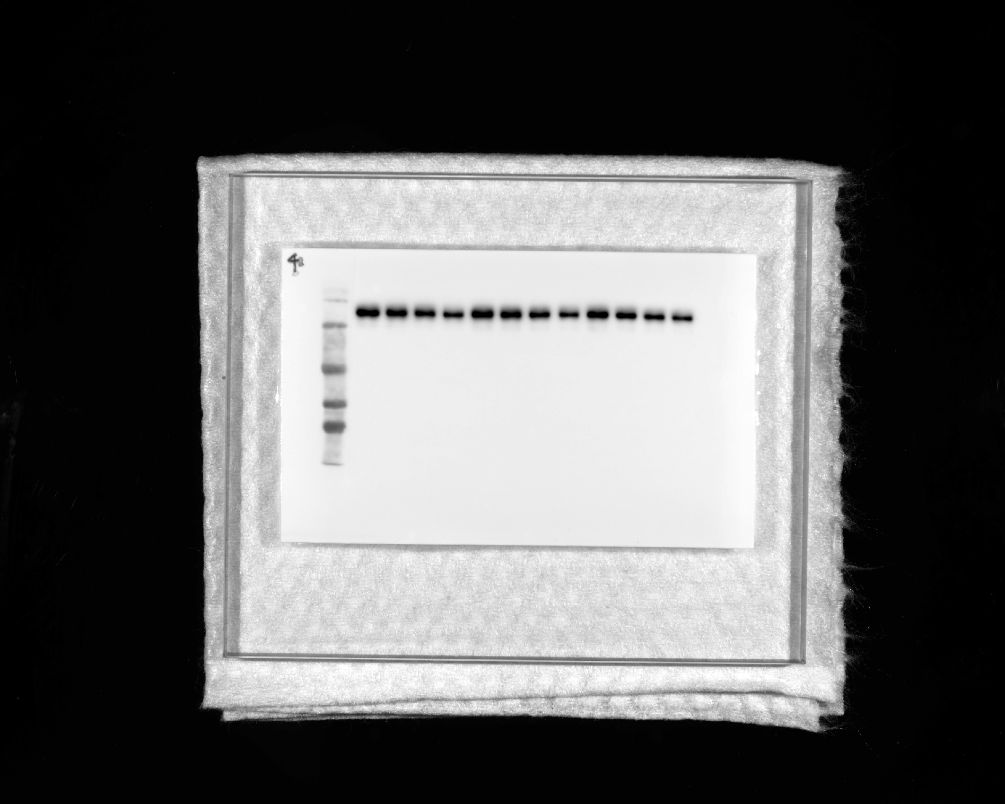 |
| PTEN  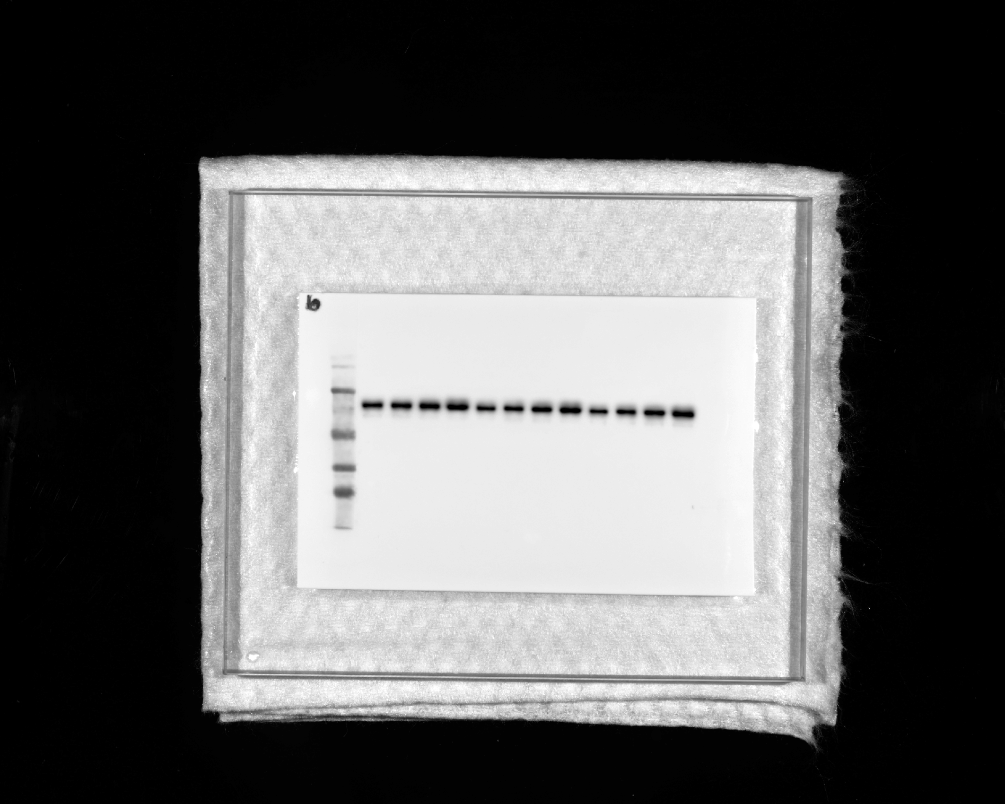  CCRF-CEM  CCRF-CEM+1μM地西他滨  CCRF-CEM+10μM地西他滨  CCRF-CEM+100μM地西他滨  CCRF-CEM  CCRF-CEM+1μM地西他滨  CCRF-CEM+10μM地西他滨  CCRF-CEM+100μM地西他滨  CCRF-CEM  CCRF-CEM+1μM地西他滨  CCRF-CEM+10μM地西他滨  CCRF-CEM+100μM地西他滨  120 kDa  100 kDa  70 kDa  50 kDa  40 kDa  30 kDa  25 kDa  14 kDa |
